# Identifying Anatomical Origins of Coexisting Oscillations in the Cortical Microcircuit

**DOI:** 10.1371/journal.pcbi.1005132

**Published:** 2016-10-13

**Authors:** Hannah Bos, Markus Diesmann, Moritz Helias

**Affiliations:** 1 Institute of Neuroscience and Medicine (INM-6) and Institute for Advanced Simulation (IAS-6) and JARA BRAIN Institute I, Jülich Research Centre, Jülich, Germany; 2 Department of Psychiatry, Psychotherapy and Psychosomatics, Medical Faculty, RWTH Aachen University, Aachen, Germany; 3 Department of Physics, Faculty 1, RWTH Aachen University, Aachen, Germany; UCL, UNITED KINGDOM

## Abstract

Oscillations are omnipresent in neural population signals, like multi-unit recordings, EEG/MEG, and the local field potential. They have been linked to the population firing rate of neurons, with individual neurons firing in a close-to-irregular fashion at low rates. Using a combination of mean-field and linear response theory we predict the spectra generated in a layered microcircuit model of V1, composed of leaky integrate-and-fire neurons and based on connectivity compiled from anatomical and electrophysiological studies. The model exhibits low- and high-*γ* oscillations visible in all populations. Since locally generated frequencies are imposed onto other populations, the origin of the oscillations cannot be deduced from the spectra. We develop an universally applicable systematic approach that identifies the anatomical circuits underlying the generation of oscillations in a given network. Based on a theoretical reduction of the dynamics, we derive a sensitivity measure resulting in a frequency-dependent connectivity map that reveals connections crucial for the peak amplitude and frequency of the observed oscillations and identifies the minimal circuit generating a given frequency. The low-*γ* peak turns out to be generated in a sub-circuit located in layer 2/3 and 4, while the high-*γ* peak emerges from the inter-neurons in layer 4. Connections within and onto layer 5 are found to regulate slow rate fluctuations. We further demonstrate how small perturbations of the crucial connections have significant impact on the population spectra, while the impairment of other connections leaves the dynamics on the population level unaltered. The study uncovers connections where mechanisms controlling the spectra of the cortical microcircuit are most effective.

## Author Summary

Recordings of brain activity show multiple coexisting oscillations. The generation of these oscillations has so far only been investigated in generic one- and two-population networks, neglecting their embedment into larger systems. We introduce a method that determines the mechanisms and sub-circuits generating oscillations in structured spiking networks. Analyzing a multi-layered model of the cortical microcircuit, we trace back characteristic oscillations to experimentally observed connectivity patterns. The approach exposes the influence of individual connections on frequency and amplitude of these oscillations and therefore reveals locations, where biological mechanisms controlling oscillations and experimental manipulations have the largest impact. The new analytical tool replaces parameter scans in computationally expensive models, guides circuit design, and can be employed to validate connectivity data.

## Introduction

Understanding the origin and properties of oscillations [see 1, for a review] is of particular interest due to their controversially discussed functional roles, such as binding of neurons into percepts and selective routing of information [reviewed in 2, esp. part VI]. Specific frequencies have been localized in different layers and linked to top-down and bottom-up processes [3, 4].

Oscillations in population signals correlate with multi-unit spiking activity [5], predominantly at high frequencies [6, 7], while firing probabilities relate to the phase of low frequency oscillations [8].

Coherent oscillations at the population level can arise from clock-like firing cells [9, 10] and more robustly [11] from irregularly firing neurons synchronizing weakly [12, 13]. Neurons in vivo tend to fire irregularly [14] and population oscillations resemble filtered noise rather than clock-like activity [15, 16]. Balanced random networks of leaky integrate-and-fire neurons in the asynchronous irregular (AI) regime can sustain such weakly synchronized oscillatory states [17] and reproduce the stochastic duration and power spectra of *γ* oscillations [18, 19].

Focusing on the network aspect, rather than on intrinsic cell properties, the PING and ING mechanisms have been suggested to underlie the generation of low- and high-*γ* frequencies [20, reviewed in 21]. Inter-neuron *γ* (ING) consists of a self-coupled inhibitory population producing an oscillation frequency primarily determined by the time course of the inhibitory postsynaptic potential (IPSP), the dynamical state of the neurons [20, 22, 10, 23] and the delays [24], constraining the generated frequency to the high-*γ* (> 70 Hz) range.

Lower-*γ* frequencies (30–70 Hz) arise from the interplay of pyramidal- and inter-neurons (PING) with the frequency determined by the dynamical state of the neurons and the connection parameters [25, 26, 27, 28]. Early network models combining ING and PING motifs [29], as well as self-coupled excitatory populations (studied later in [30]), enabled the phenomenological study of *γ* oscillations. The two mechanisms were originally formulated for the fully synchronized regime and the analytical treatment of weakly synchronizing networks is restricted to at most two populations [17, 31], neglecting the variety of dynamical states of neuronal populations embedded in a larger circuitry. Modeling studies considering neurons of various level of detail assess the link between network structure and induced oscillations [32]. Experiments find specific frequencies at different depths of the layered cortex [33], while these locations in the tissue are characterized by distinct connectivity patterns [34]. Pronounced slow oscillations (< 1 Hz) are found in deeper layers, such as layer 5 [35, 36], and hypotheses regarding their origin range from intrinsic cell mechanisms [37] to network phenomena [38, 39]. In contrast, fast oscillations in the *γ* and high-*γ* range are primarily observed in the upper layers [40, 41] and show different phase relationships than the *γ* oscillations in the lower layers [42].

To the best of our knowledge, theoretical descriptions of coexisting oscillations requiring complicated network structures, as well as a method identifying these structures in a given circuit have not yet been established. The present work sheds light on the influence of sub-circuits integrated in larger networks and the properties of individual connections relevant for the emergence of specific oscillations.

## Results

### Population rate spectra in simulations of the microcircuit

**Fig 1:**
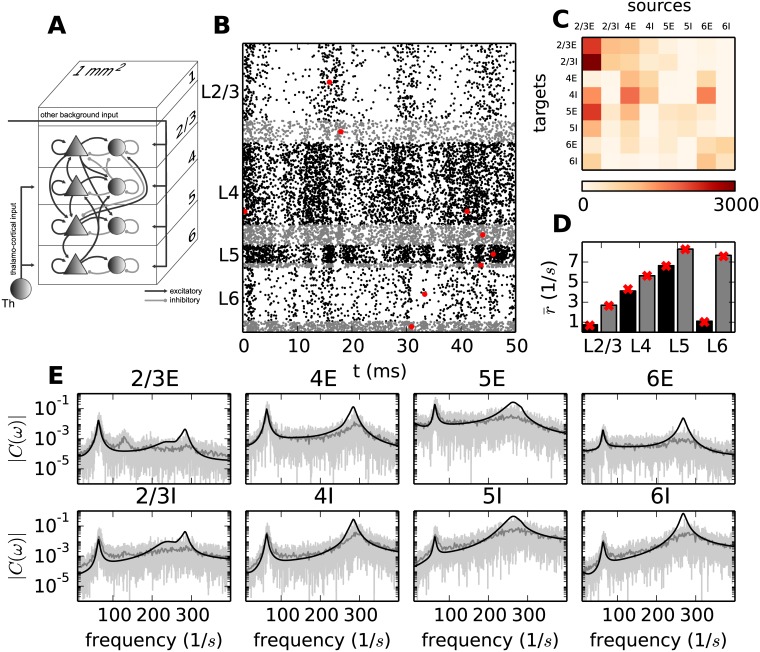
Activity in the microcircuit model. **A** Sketch of the layered connectivity structure of the model adapted from Fig 1a of Potjans and Diesmann [34]. **B** Dot plot marking the spike times of all neurons in a 50 ms segment of a direct simulation of the model in A. Black dots denote spike times of excitatory and gray dots of inhibitory neurons. The red dots mark the firing times of one particular neuron per population. **C** Average number of indegrees. **D** Average firing rates of neurons for each population obtained by simulation (black bars: excitatory population, gray bars: inhibitory population) and theoretical predictions (red crosses). **E** Raw spectra extracted from a simulation of 10 s by the Fast Fourier Transform (FFT) algorithm using a binning of 1 ms (light gray curves) and averaged over 500 ms windows (gray curves) and the analytical prediction (black curves). The top row shows the spectra in the excitatory and the bottom row the spectra in the inhibitory populations.

The multi-layered spiking cortical network model used throughout this study was introduced by Potjans and Diesmann [34]. The model is composed of four layers (L2/3, L4, L5 and L6), each layer containing an excitatory and an inhibitory population of neurons (Fig 1A). The number of neurons in each population, as well as the number of connections between and within populations are extracted from experimental data sets [for a full list of references see Table 1 of ref. 34]. Combining the data yields the 8 × 8-dimensional indegree matrix **K** (Fig 1C), where the element *K*_*ij*_ describes the number of connections from population *j* to population *i*. Given the total number of connections between populations, the pre- and postsynaptic neurons of the individual connections are drawn randomly. Each population receives additional Poisson spike trains resembling the activity of other brain regions. Potjans and Diesmann show by simulations that the population firing rates generated within the model reproduce those observed in experiments [43, 44]. The neurons are modeled by leaky integrate-and-fire (LIF) dynamics with exponentially decaying synaptic currents:

$$\begin{array}{ccc} \tau_{m}\frac{dV_{ki}\left(t\right)}{dt} & = & -V_{ki}\left(t\right)+RI_{ki}\left(t\right)\\ \tau_{s}\frac{dI_{ki}\left(t\right)}{dt} & = & -I_{ki}\left(t\right)+\tau_{s}\sum_{l=1}^{N}\sum_{j=1}^{M_{l}}w_{ki,lj}\sum_{n}\delta \left(t-t_{lj}^{n}-d_{ki,lj}\right)+\tau_{s}\sum_{j=1}^{M_{ext}}w_{ki,j}\sum_{n}\delta \left(t-t_{j}^{n}\right). \end{array}$$ (1)

Here *V*_*ki*_(*t*) describes the membrane potential of the *i*-th neuron in the *k*-th population and *I*_*ki*_(*t*) the incoming synaptic current to this neuron. *R* denotes the resistance of the membrane and *τ*_*m*_ the membrane time constant, *τ*_*s*_ the synaptic time constant, *w* and *d* the weight and delay associated to the incoming events, and $t_{lj}^{n}$ the time of the *n*-th spike of neuron *j* in population *l*. The number of populations is given by *N* and the number of neurons in the *l*-th population is denoted by *M*_*l*_. In addition to the spikes from within the network, each neuron receives spikes from *M*_*ext*_ external sources representing the input of other brain regions by spike times $t_{j}^{n}$ drawn from Poisson processes with rates specified in [34].

Simulating the microcircuit model, we first reproduce the dynamics observed in [34] and additionally investigate the correlation structure of the system. After simulating the circuit for *T* = 10 s with a time resolution of 0.01 ms, we observe averaged population specific firing rates between 0.9 Hz and 8.6 Hz, which reflect tendencies of population specific firing rates in experimental data [34]. The average coefficients of variation (CV) of the neurons are around 0.55 for the populations with low firing rates (2/3E and 6E) and around 0.8 for the other populations, characterizing the spike trains of individual neurons as irregular. The irregular nature of the spike trains is underlined by the raster plot (Fig 1B) showing all spike times in a 50 ms segment. The vertical stripes visible in the spike times of some populations suggest a certain degree of synchrony in the activity of the neurons on the population level. However, this regularity is barely exhibited on the single neuron level, which participate only in a fraction of the cycles (red dots in Fig 1B). A comparison of the auto-correlations of the individual neurons with the auto-correlations of the population shows that the population spectrum is dominated by the cross-correlations and the contribution of the individual auto-correlations is negligible in agreement with Tetzlaff et al. [45].

### Mean-field and linear response description of the microcircuit

**Fig 2:**
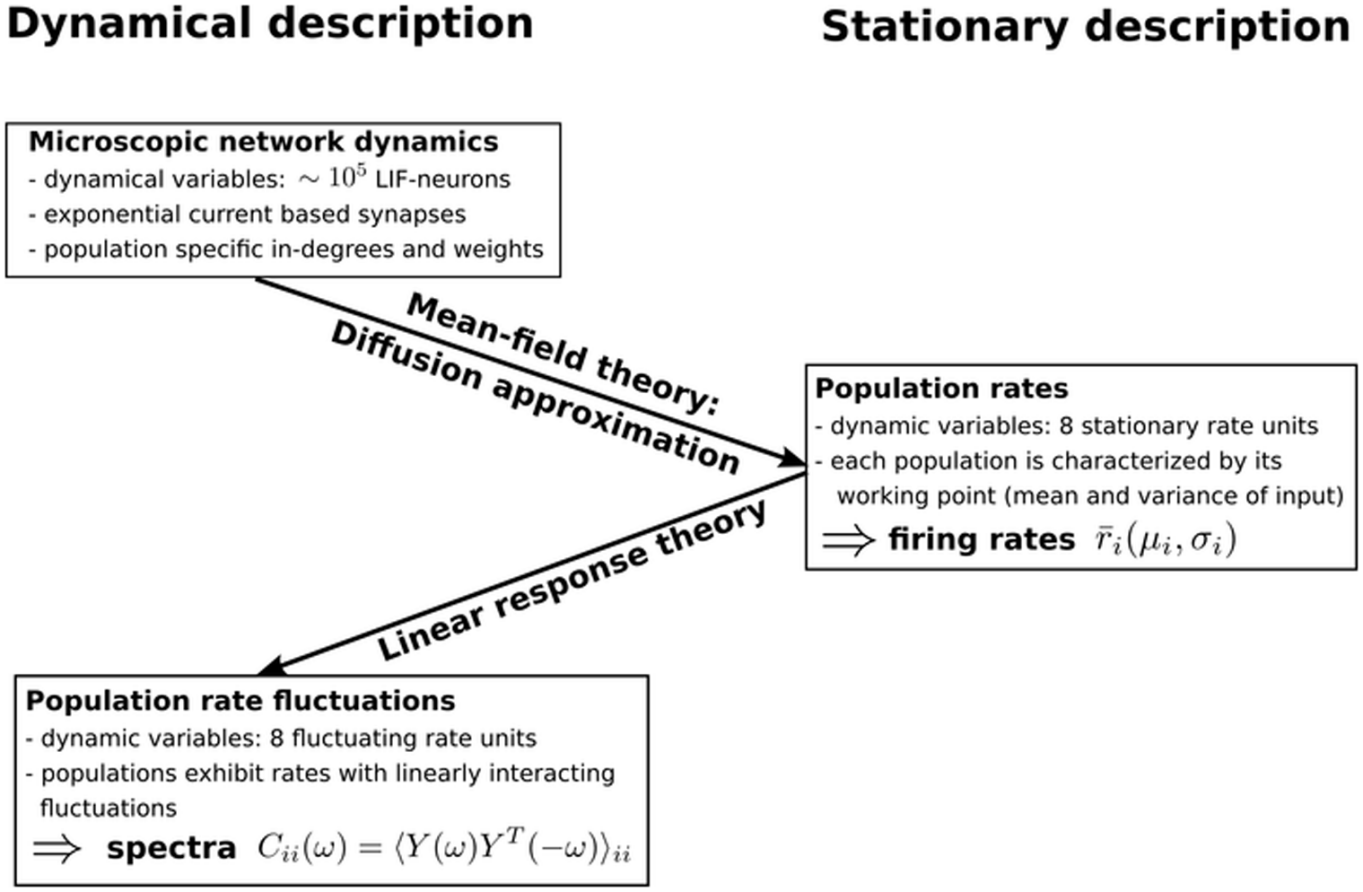
Sketch of the mean-field reduction and linear response theory of fluctuations. Graphical representation of the two-step reduction carried out when deriving the description of fluctuations in recurrent networks. First, the working point of each population, determined by the mean and variance of its input is established by means of diffusion approximation. The working point suffices to predict the stationary firing rates of the populations, which follow from the self-consistent solution of a set of mean-field equations. This step constitutes a dimensionality reduction from two dynamic variables for each of the *N* = 77169 neurons, to one dynamic variable for each of the eight populations. Second, the dynamical responses of the populations are approximated by linear response theory around the stationary solution. The fluctuations of the population activities can subsequently be mapped to a linear rate model. The derived description of the rate fluctuations yields the population rate spectra, as described in “*Fluctuation dynamics*”.

The description of fluctuations in spiking networks deployed in this study proceeds in two steps, as summarized in Fig 2. In the first step, we use mean-field theory for spiking neurons [46] to determine the stationary state of the network, i.e. the time-independent averaged firing rates of each population. In particular, the original high-dimensional system, composed of the two dynamical variables in Eq (1) for each of the *N* neurons, is reduced by means of diffusion approximation. Here we exploit the fact that each neuron receives a large number of inputs, with each incoming impulse eliciting only a small deviation of the membrane potential, which allows us to treat inputs linearly. Assuming additionally that correlations between the populations are only displayed by fluctuations around a stationary state but that the stationary rates of the populations can be obtained by neglecting these correlations, enables us to describe the total synaptic input to population *i* as Gaussian white noise characterized by mean and variance [46, 13]

$$\begin{array}{c} \mu_{i}=\tau_{m}w\left(\sum_{j\in E}K_{ij}{\bar{r}}_{j}-g\sum_{j\in I}K_{ij}{\bar{r}}_{j}+K_{\text{ext},i}r_{\text{ext}}\right)\\ \sigma_{i}^{2}=\tau_{m}w^{2}\left(\sum_{j\in E}K_{ij}{\bar{r}}_{j}+g^{2}\sum_{j\in I}K_{ij}{\bar{r}}_{j}+K_{\text{ext},i}r_{\text{ext}}\right). \end{array}$$ (2)

Here *w* denotes the synaptic weight, *K*_*ij*_ the indegrees from population *j* to population *i*, *g* the ratio between excitation and inhibition and ${\bar{r}}_{j}$ the yet to be determined stationary firing rate of the *j*-th population. The mean and variance of the input current to a population is referred to as its working point. In this approximation, the stationary firing rates are a function of the working point, yielding the self-consistency equation

$${\bar{r}}_{i}=\nu_{i}\left(\mu_{i},\sigma_{i}\right)=\nu_{i}\left(\mu_{i}\left(\bar{r}\right),\sigma_{i}\left(\bar{r}\right)\right)$$ (3)

where the vector $\bar{r}=\left({\bar{r}}_{1},...,{\bar{r}}_{N}\right)$ denotes the stationary rates of the *N* populations and the function *ν*(*μ*, *σ*) is derived from the stationary solution of the Fokker-Planck equation for the probability distribution of the membrane potentials (see equation 4.33 in [47]). Fig 1D shows that this approximation suffices to predict the rates in the microcircuit model. In the second step, we analyze how small fluctuations around this stationary state propagate within the network and how their dynamics can be mapped to that of a linear rate model [48, 49]. To this end we employ linear response theory, applied to the leaky integrate-and-fire model, using an extension of the work of [13] and [12] to colored synaptic noise [50]. We here summarize the main results of the mapping of the dynamics of the fluctuations, referring the reader to [49] for the detailed derivations. The reduction allows for a self-consistent dynamical description of the fluctuations of the population rates. The output rate (left-hand side) relates to the input (right-hand side) via

$$R\left(\omega \right)={\tilde{M}}_{d}\left(\omega \right)Y\left(\omega \right),\;\text{with}\;Y\left(\omega \right)=R\left(\omega \right)+X\left(\omega \right).$$ (4)

Here, **R**(*ω*) denotes the eight dimensional rate vector in Fourier space. The realization of the instantaneous rate as a spike train is approximated by Poisson statistics, giving rise to the noise term **X**(*ω*). The input to the populations is weighted by the connectivity and filtered by the transfer function of the populations (summarized in the effective connectivity matrix ${\tilde{M}}_{d}\left(\omega \right)$). The formulation of the rate dynamics yields predictions for the population rate spectra (for further details see “*Fluctuation dynamics*”). Fig 1E shows that the low-*γ* peak around 64 Hz, visible in the spectra of all populations, is well predicted by this theory. As suggested by the regularly occurring vertical stripes in Fig 1B, we observe a high-frequency peak in all populations varying from 235 Hz to 303 Hz, which is most prominent in layer 4. It can be shown that in the context of the low-*γ* peak, the network is in the asynchronous irregular (AI) regime [12], where the linear response theory suffices to describe the noise fluctuations. However, on the time scale of the high-frequency peak the network verges on the border of the synchronous irregular state (SI), resulting in deviations of the theoretical prediction from the observed oscillations. The frequency of the fast oscillations depends strongly and inversely on the synaptic time constants, which are small in the present model (*τ*_syn_ = 0.5 ms). Therefore the frequency would be considerably lower for longer time constants (see “*The high-γ peak*”). The high-frequency peak will therefore, in the following, be referred to as the high-*γ* peak.

Given the density of connections in the circuit (Fig 1C), the similarity of the spectra hints at the oscillation being generated in a sub-circuit of the microcircuit and subsequently imposed onto all populations. This prevents the identification of the sub-circuitry generating the oscillation on the basis of the spectra. Thus the analytical tools developed in our study up to this point enable the prediction of the population firing rate spectra Eq (18), but do not allow for the inspection of the underlying circuits determining the characteristics of the spectra.

### Activity modes of the microcircuit

Rate profiles have been observed to vary across cortical layers [44], with inhibitory neurons displaying higher rates than excitatory neurons [for a review see 34]. As a result of the population specific rates, each population processes the afferent time-dependent activity with its specific temporal filter, called transfer function in systems theory [51]. Here, we summarize how peaks at different frequencies in the spectra can be associated with the activity of coexisting dynamical modes, which can be found by a linear basis transformation of the rate fluctuations, as described in “*Dynamical modes*”. Intuitively, a mode describes the tendency of a set of neuronal populations to co-fluctuate with a fixed relationship between their amplitudes and relative phases. Subsequently we discuss how population specific transfer functions result in the commingling of modes across frequencies and therefore hinder the analytical tractability of the anatomical origins of the oscillations.

Considering the linearity of the relation between input to the populations and the resulting output rate Eq (4) (for details see Eq (14) and the derivation in “*Fluctuation dynamics*”), the influence of the connectivity on the rate dynamics and thus the shape of the spectrum appears to be straightforwardly investigated by applying tools from linear algebra to the effective connectivity matrix, which is defined by the element-wise product (Hadamard product) of the anatomical connectivity Eq (13), determined by connection weights, indegrees and the population specific transfer functions ${\tilde{M}}_{d,ij}\left(\omega \right)=M_{ij}^{A}H_{d,ij}\left(\omega \right)$. Different modes of the circuit are found by eigenvalue decomposition of the effective connectivity matrix ${\tilde{M}}_{d,ij}\left(\omega \right)$ and are, in the following, therefore referred to as eigenmodes.

**Fig 3:**
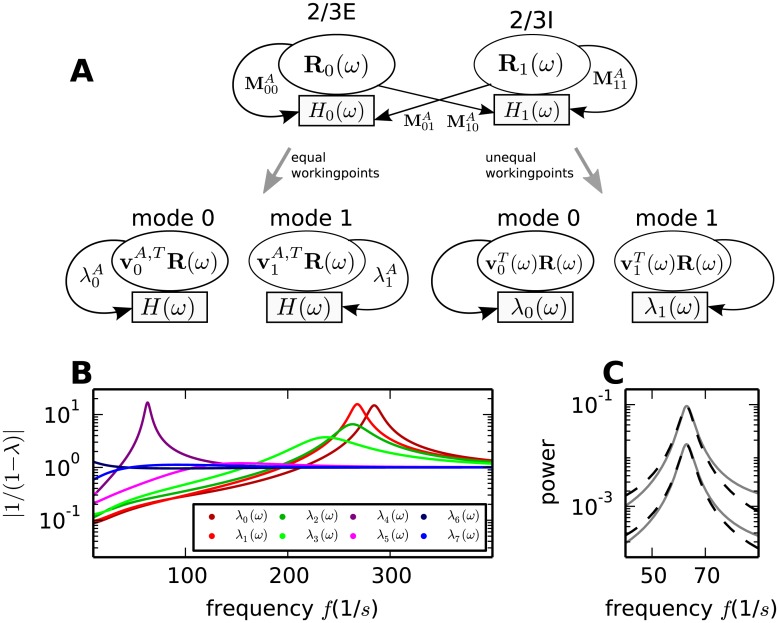
Decomposition of activity into independent modes. **A** Visualization of the equation describing the rate fluctuations obtained by linear response theory, as given in Eq (4). The illustration depicts the linear transformation (thick gray arrows) of the original circuit (top) into the basis of eigenvectors of the effective connectivity matrix, yielding a circuit containing dynamic modes which solely couple to themselves (bottom) (see “*Dynamical modes*”). In the simplified system depicted on the left, the transfer functions of all populations are equal, while the populations shown on the right are in different dynamical states. For simplicity only layer 2/3 is shown. *R*_*i*_(*ω*) denotes the rate of the *i*-th population. In the original basis (top), the rate is propagated along the arrows to the target population, where it triggers a response weighted by the connection property $M_{ji}^{A}$ and filtered by the transfer function of the receiving population *H*_*j*_(*ω*). In the new coordinate system (bottom), the activity of the modes is described by the projection of the former rate vector onto eigenvectors **v**_*i*_(*ω*) of the effective connectivity matrix. The former sequence of weighting by the anatomical connectivity and filtering by the transfer functions of the populations is combined in the mapping onto the corresponding eigenmode. The eigenvalue of the mode λ_*i*_(*ω*) serves as the transfer function of the mode. **B** Frequency dependence of the factors |1/(1 − λ_*i*_(*ω*))| determining the global shape of the spectrum. **C** Spectra of the excitatory populations of layer 2/3 and 4 (solid gray curves) and the approximate spectra (dashed black curves) obtained by substituting the projection onto the dominant eigenmode for the effective connectivity matrix.

Their dynamical behavior is linked to the frequency dependence of the corresponding eigenvalues as shown in Fig 3B and discussed in “*Dynamical modes*”. Indeed, we observe that the term *p*(*ω*) = |1/(1 − λ_*i*_(*ω*))| exhibits a peak at around 60 Hz with a shape reminiscent of the peaks in the theoretically predicted spectra as well as in the spectra observed in simulations (Fig 1). This similarity is not surprising, since the inverse of the distance of the eigenvalue to one contributes to the spectrum of all populations Eq (22). At higher frequencies the eigenvalues of four modes produce a maximum with the dominant mode being largest at 275 Hz, corresponding to the high-*γ* peak in Fig 1E. All modes but one exhibit small terms *p*(*ω*) for low frequencies. The involvement of the mode that corresponds to the large values of *p*(*ω*) at low frequencies in the generation of slow rate fluctuations is discussed in the following sections.

At peak frequencies the dynamics of the circuit can be approximated by the dynamics of the dominant mode Eq (31). Results for the spectra in the excitatory populations of layer 2/3 and 4 are shown in Fig 3C. The reduced circuitry suffices to approximate the spectrum around the low-*γ* peak, but for lower and in particular higher frequencies the absence of contributions of the remaining modes becomes apparent.

Since the dynamics in the vicinity of a peak are well approximated by the dominant mode, the question arises as to how much information about the minimal anatomical circuit producing the same oscillation is contained in the mode representation. As discussed in “*Dynamical modes*” and illustrated in Fig 3A, a frequency independent projection from the dynamics of the full circuit to the dynamical modes is only attainable if all populations have the same firing rate and transfer function. In this case each dynamical mode can be traced back to one particular set of anatomical connections which does not influence the dynamics of the other modes.

Heterogeneous rates and response properties of the populations result in dynamical modes composed of different anatomical connections at different frequencies. Thus the mapping between a set of anatomical connections and a dynamical mode is limited to one particular frequency, while the same set of connections might influence other modes at other frequencies. The representation of the activity modes determining the characteristics of the spectra is hence frequency dependent. Therefore the eigenmode cannot straightforwardly be mapped to the relevant anatomical connection as in the case of networks with populations in homogeneous dynamical states.

### Modes governing the spectrum

In this section we provide an intuitive understanding of how the dynamics of the eigenvalues determine the spectra as well as how the eigenvalues originate from the connectivity of the individual layers and are shaped by the connections between the layers. Readers primarily interested in the final method used to detect the origin of the oscillations may skip this section.

**Fig 4:**
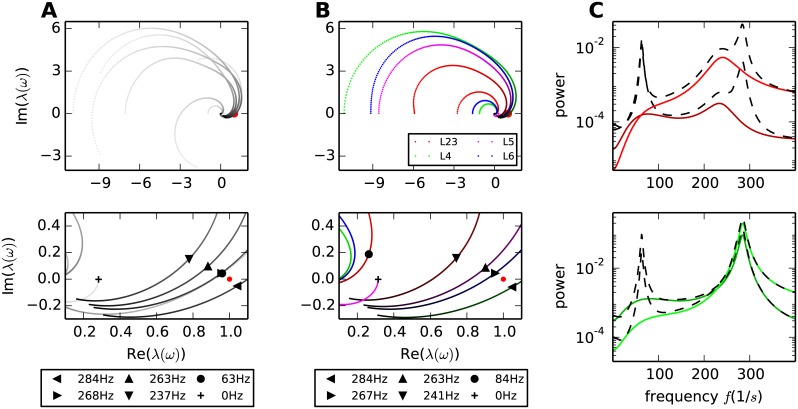
Frequency-dependence of modes in the original circuit and in isolated layers. **A** Trajectories of the eight eigenvalues of the microcircuit in the complex plain (upper panel) parameterized from 0 Hz (light) to 400 Hz (dark) and an enlargement (lower panel) of the area around one (red dot). The point where a trajectory comes closest to one is marked by a cross and the legend shows the corresponding frequency. **B** Eigenvalue trajectories of the isolated layers with same parameterization as in A. **C** Spectra of populations 2/3E (dark red), 2/3I (light red), 4E (dark green) and 4I (light green) in the isolated layers (solid curves) and in the original circuit (dashed curves).

The dynamics of the eigenmodes are mainly described by the eigenvalues. The frequency dependence of the complex-valued eigenvalues, termed “trajectories” in the following, is visualized in parametric plots (Fig 4A). These plots, also known as Nyquist plots [51, Chapter 11], allow for the simultaneous investigation of the real and imaginary part of the eigenvalue. Fig 4A shows the trajectories of the eight eigenvalues of the microcircuit in the complex plane up to 400 Hz. The movement of a trajectory is reminiscent of a spiral starting at the eigenvalue of the effective connectivity matrix at zero frequency and spiraling clockwise towards zero with increasing frequency. Fig 3B together with the final expression for the spectrum Eq (22) shows that the amplitude of the spectrum increases the closer an eigenvalue approaches the value one and decreases the further it moves away. The spectrum diverges if the eigenvalue assumes one, which will therefore be referred to as the critical value one. The implications of this critical value for the stability of the circuit are discussed in more detail in “*Stability of the dynamical modes*”. All trajectories eventually converge to zero, reflecting that the modes cannot follow very high frequencies. Hence the term *p*(*ω*) = 1/|(1 − λ_*i*_(*ω*))|, which contributes considerably to the shape of the spectrum Eq (22), converges to one for high frequencies. The eigenvalue trajectories λ_*i*_(*ω*) in the microcircuit are typically continuous and spin clockwise for all frequencies. Hence, if a trajectory reaches the sector of positive real parts it will, in the general case, for at least one frequency *ω*_*p*_ assume a closer distance to one than in the large frequency limit. At this frequency the term *p*(*ω*_*p*_) assumes larger values than one, yielding a peak in the spectrum.

The eigenvalues can be interpreted as the transfer function of the modes (Fig 3A), which offers an intuition for the impact of synaptic parameters the spectra, as outlined in “*Eigenvalue trajectories are transfer functions of the dynamical modes*”. From the shape of the transfer functions corresponding to the populations Eq (15) we deduce that small delays slow down the spinning of the trajectories. The eigenvalue therefore passes by one at a high frequency resulting in rapid oscillations. Long delays accelerate the trajectories yielding slow oscillations. However, the longer the delay the larger the radius of the trajectories. Once the eigenvalue assumes a real part larger than one at the frequency where it passes closest to the critical value one the mode produces activity in the SI regime. The transition from the regime where the real part of the eigenvalue is smaller than one to the regime where the real part is larger than one is characterized by a change in stability. Below one, the dynamics relaxes back to the stationary rates in an oscillatory fashion determined by the frequency at which the distance to one is minimal with a damping related to this minimal distance. Nevertheless, since the noise propagating in the system repeatedly excites these oscillatory decaying modes, the oscillations are visible in the population rate spectra. Altering the parameters of the circuit such that the eigenvalue assumes the value one for some frequency *ω*, the system (defined by Eq (4) in the Fourier and by Eq (11) in the time domain) transits from damped to amplified oscillatory modes (as discussed in more detail in “*Stability of the dynamical modes*”). Growing modes are restrained by the non-linearities of the neurons. Hence, the point at which one eigenvalue assumes the value one characterizes the passage of the corresponding mode dynamics from oscillatory decaying rate perturbations to the onset of sustained oscillations. If the eigenvalue assumes real values larger than one, the equivalent linear rate system is unstable and displays an irresistibly growing oscillatory mode. The system of LIF model neurons, however, provides stabilizing non-linearities (due to the reset after firing and the fact that the rates of the neurons cannot become negative) which prevent the dynamics from exploding. Since the presented theoretical framework does not account for non-linearities, it is limited to predicting the tendencies of the spectra in the latter case as visible in the high frequency peak in Fig 1E.

The radius of the trajectory is compressed by widely distributed delays Eq (15), allowing for the production of slower oscillations caused by long delays without destabilization of the dynamics.

In order to analyze the dynamics originating in the individual layers we calculate the eigenvalue trajectories of the isolated layers. The input of other layers is provided by means of Poisson spike trains. This approach holds the dynamic state of the populations constant while neglecting the correlations induced by the input from other layers. Hence the collective dynamics emerging locally in each layer can be analyzed. The eigenvalue trajectories corresponding to the isolated layers are displayed in Fig 4B.

Since the connectivity within the layers is more pronounced than the connectivity between the layers, we can deduce the origin of some of the eigenvalue trajectories by comparing their characteristics with the characteristics of the trajectories in the original circuit (Fig 4A and 4B). In isolation, layer 2/3 produces an eigenvalue which passes closest to one at 87 Hz. Since the distance to one is large, the eigenvalue trajectory produces only a small peak in the spectrum of layer 2/3 (Fig 4C). Layer 4 in isolation does not generate a low-*γ* peak (Fig 4C). Connecting the layers, the eigenvalue trajectories of layers 2/3 and 4 mix and produce a trajectory with a positive imaginary offset and a sufficiently small real-valued starting point to pass close by one at a relatively low frequency (60 Hz), resulting in a peak in the spectra of all populations.

In the high frequency range we observe four trajectories originating in the four layers passing close by one. The course of the trajectories is only mildly impacted when integrated into the full circuit. Therefore we predict a high frequency peak visible in the populations, even in the isolated layers. However, considering the spectra in layer 2/3 and 4 we observe that the high frequency peak in layer 4 matches the peak observed in the full circuit, whereas the peak produced in layer 2/3 is of smaller frequency and amplitude. Therefore we can already conclude that the high-*γ* peak originates in layer 4 and is propagated to the other layers when embedded in the full circuit.

In addition to the origin of the oscillation, the eigenvalue trajectories shed light on the stability of the circuit and the associated oscillations. Following the classification of Brunel [54], the dynamics of a mode transits from the AI to the SI regime via a Hopf bifurcation if there is a frequency at which the corresponding eigenvalue equals one. The dynamics is in the AI regime, i.e. the system possesses a stable fixed point of the rates, if the closest encounter of the corresponding trajectory with the critical value one is located on the left of the latter Fig 4C (see also “*Stability of the dynamical modes*”). Here, temporal structure in the firing rates arises from oscillatory, but decaying perturbations, which are continuously excited by the noise generated within the system. The damping factor of decaying perturbations grows with the distance of the eigenvalue from one, while the amplitude of the peak in the spectrum diminishes. In addition, Brunel et al. [13] show that networks of LIF-model neurons close to a bifurcation point are stabilized by the non-linearity of the neuron dynamics. Hence, perturbations decaying with low-*γ* frequency are strongly damped. The assumption of independence on the level of individual neurons, which underlies the mean-field approximation in the first step of our framework, is therefore justified. This is reflected by the match of the theoretical prediction of the low-*γ* peak with the peak observed in simulations of the microcircuit (Fig 1). Fig 3C shows that one of the eigenvalues associated to the high-*γ* peak lies to the right of one (corresponding to the SI regime in [52]), which, in a linear system, would yield growing oscillatory perturbations. However, in networks of LIF-model neurons these oscillations are tamed by the non-linearity of the neurons. Accordingly, for the high-*γ* peak, the theoretical framework only suffices to predict the tendencies of the peak in the spectrum (Fig 1).

In summary, employing a combination of mean-field and linear response theory we consider the dynamical contributions of the individual layers. In an iterative fashion we narrowed down the origin of the high-*γ* peak to layer 4. In addition we find indication for the low-*γ* peak being shaped in layers 2/3 and 4. Fig 3C and 3D shows the spectra of distinct layers of a network that can in isolation be described as band-pass filters. However, the connections between the layers contribute strongly to the generated oscillation. This is reflected in the fact that the filter properties of the layers change once inter-layer connections are considered showing that the network needs to be considered in its entirety to predict the spectra. Hence this iterative approach provides insight regarding the structures shaping the oscillations, but is potentially time-consuming especially when circuits with large numbers of populations are considered.

### Sensitivity measure

Here we set out to develop a systematic approach identifying the connections involved in the generation of the frequency peaks. So far we identified the eigenmode responsible for the peak generation by considering its proximity to the critical value one. Since the distance of the eigenvalue at peak frequency to one scales the amplitude of the peak in the power spectrum, we can define important anatomical connections as connections the eigenvalue is particularly sensitive to. In the following, the eigenvalue evaluated at peak frequency is referred to as the critical eigenvalue. Mathematically sensitivity is assessed by introducing a small perturbation to the indegree matrix at the connection from the *l*-th to the *k*-th population

$${\hat{K}}_{ij}\left(\alpha_{kl}\right)=\left(1+\alpha_{kl}\delta_{ki}\delta_{lj}\right)K_{ij}.$$ (5)

Thus, depending on the sign of the perturbation, the *kl*-th element of the indegree matrix is decreased or increased by the fraction *α*_*kl*_. Before we continue the formal perturbation analysis let us briefly look at the interpretation of such a perturbation in the context of our theoretical framework. Our aim is to analyze the contribution of the connection from population *j* to population *i* on the fluctuations of activity expressed by the spectra. The linear response theory treats fluctuations of network activity around the stationary state up to linear order. The stationary state itself (determining the firing rates) as well as the transfer functions of the populations are in this approximation therefore not effected by the activity fluctuations. We hence study the affect of the connections while conserving their embedment in the full circuit. This separation of the contribution of connections to the correlations from their contribution to the stationary state can be realized in direct simulations by counteracting the perturbation in the number of synapses within the circuit by an adjustment of the external input to the populations. In this way the stationary properties remain fixed since the mean and variance of the input to the neurons are unaltered. However, the correlation structure generally changes since the connections within the circuit, which induce correlations due to the specificity of the connectivity, are substituted by external connections providing uncorrelated input.

The perturbed effective connectivity matrix is obtained by inserting the new indegrees into Eqs (13) and (12)

$${\hat{M}}_{ij}\left(\alpha_{kl}\right)=\left(1+\alpha_{kl}\delta_{ki}\delta_{lj}\right){\tilde{M}}_{ij}.$$ (6)

We define the sensitivity measure *Z*_*kl*_(*ω*) as the derivative of the critical eigenvalue of the perturbed system at frequency *ω* with respect to the perturbation [53]

$$\begin{array}{c} Z_{kl}≔\frac{\partial {\hat{\lambda }}_{c}\left(\alpha_{kl}\right)}{\partial \alpha_{kl}}{|}_{\alpha_{kl}=0}=\frac{{\hat{v}}_{c}^{T}\left(\alpha_{kl}\right)\frac{\partial \hat{M}\left(\alpha_{kl}\right)}{\partial \alpha_{kl}}{\hat{u}}_{c}\left(\alpha_{kl}\right)}{{\hat{v}}_{c}^{T}\left(\alpha_{kl}\right){\hat{u}}_{c}\left(\alpha_{kl}\right)}{|}_{\alpha_{kl}=0}\\ =\frac{v_{c,k}{\tilde{M}}_{kl}u_{c,l}}{v_{c}^{T}u_{c}}, \end{array}$$ (7)

where ${\tilde{M}}_{kl}$ is the *kl*-th element of the effective connectivity matrix and $v_{c}^{T}$, **u**_*c*_ are its left and right eigenvectors corresponding to the critical mode. For brevity, the frequency dependence of the matrix and the eigenvectors is omitted. The elements of the matrix **Z**(*ω*) describe the direction and amplitude of the shift of the critical eigenvalue after perturbing the indegrees of the corresponding connections.

**Fig 5:**
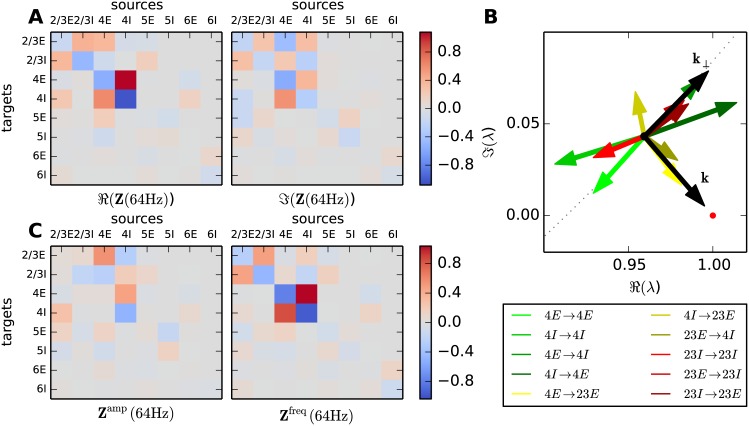
Sensitivity of oscillations to changes in connectivity. **A** Real (left panel) and imaginary part (right panel) of the sensitivity measure Eq (7) (color coded; gray: insensitive, red: positive, blue: negative) evaluated at peak frequency 64 Hz. Each matrix element corresponds to one connection in the microcircuit. **B** A selection of the most prominent matrix elements (legend) of the sensitivity measure at 64 Hz visualized as vectors in the complex plane. The red vectors are associated with connections in layer 2/3, the green vectors with connections in layer 4 and the yellow vectors with the connection between layers 2/3 and 4. The red dot denotes the critical value one. The black vector **k** starts from the critical eigenvalue and points towards one. The vector **k**_⊥_ denotes the direction perpendicular to **k**. The gray dots mark the trajectory of the critical eigenvalue parameterized by frequency. **C** Sensitivity measure in rotated coordinates separating the influence of connections on peak amplitude (left panel) and frequency (right panel), otherwise same display as in A.

The frequency dependence of the perturbed eigenvalue can be linearly approximated by

$$\hat{\lambda }\left(\alpha_{kl},\omega \right)≃\lambda \left(\omega \right)+Z_{kl}\left(\omega \right)\alpha_{kl},$$ (8)

which describes the displacement of the eigenvalue to linear order after perturbing the *kl*-th element of the indegree matrix. Hence the sensitivity measure evaluated at peak frequency exhibits large entries for connections having a strong influence on the position of the critical eigenvalue. Fig 5A shows the real and imaginary part of the sensitivity measure **Z** evaluated at 64 Hz. The influence of the individual elements on the eigenvalues can be visualized in the complex plane (Fig 5B). Given the inverse proportionality of the peak height to the distance of the eigenvalue to one Eq (22), a perturbation in a connection causing a shift of the eigenvalue towards or away from one results in an increased or decreased peak amplitude in the spectrum. If the perturbation causes a shift of the trajectory purely in the direction of one, the trajectory will pass by one at approximately the same frequency leaving the position of the peak in the spectrum unaltered. This direction is labeled by the vector **k** in Fig 5B

$$k=\left(1-ℜ\left(\lambda_{c}\right),ℑ\left(\lambda_{c}\right)\right)/\sqrt{{\left(1-ℜ\left(\lambda_{c}\right)\right)}^{2}+ℑ{\left(\lambda_{c}\right)}^{2}}.$$

A perturbation resulting in a shift of the critical eigenvalue along the perpendicular direction **k**_⊥_

$$k_{⊥}=\left(-ℑ\left(\lambda_{c}\right),1-ℜ\left(\lambda_{c}\right)\right)/\sqrt{{\left(1-ℜ\left(\lambda_{c}\right)\right)}^{2}+ℑ{\left(\lambda_{c}\right)}^{2}}$$

alters the trajectory such that it passes closest to one at a lower or higher frequency while conserving the height of the peak. This suggests a basis transformation of the complex sensitivity measure to the coordinate system spanned by the two vectors **k** and **k**_⊥_:

$$Z_{ij}^{\text{amp}}=\left(ℜ\left(Z_{ij}\right),ℑ\left(Z_{ij}\right)\right)k^{T}\;,\;Z_{ij}^{\text{freq}}=\left(ℜ\left(Z_{ij}\right),ℑ\left(Z_{ij}\right)\right)k_{⊥}^{T}.$$ (9)

The resulting matrices **Z**^amp^(*ω*) = **Z**^**k**^(*ω*) and $Z^{\text{freq}}\left(\omega \right)=Z^{k_{⊥}}\left(\omega \right)$ (shown in Fig 5C) determine the impact of the connections on amplitude and frequency of the peak.

### The low-*γ* peak

The sensitivity measure (Fig 5C) exhibits large entries in the sub-circuit composed of layer 2/3 and 4. The finding of layer 2/3 and 4 being involved in the generation of the 64 Hz oscillation is in agreement with insights gained from the eigenvalue trajectories in the previous section. We observe that the amplitude of the peak is mostly determined by connections between layers 2/3 and 4, as well as inhibitory connections within layer 4. The connections dominating the amplitude of the peak originate mostly in populations 4I, 4E, and 2/3E. The frequency, on the other hand, is shaped by the connections within the layers, with connections within layer 4 having larger impacts than connections in layer 2/3. The connection from 2/3E to 4I is the only connection from layer 2/3 to layer 4 contributing to the amplitude of the peak. Therefore this connection closes the dynamic loop between layer 2/3 and layer 4. Its role in the generation of the oscillation is discussed in the following sections. Other connections contributing to the amplitude of the peak originate and terminate in 5E.

### The high-*γ* peak

**Fig 6:**
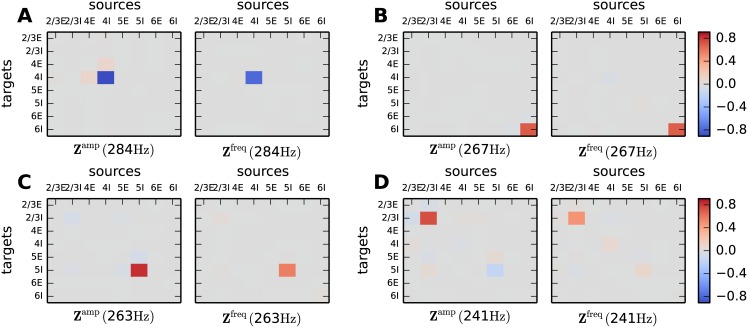
Connections relevant for high frequency oscillations. Sensitivity measure evaluated at the peak frequency of the four dominant modes in the high-*γ* regime (**A**–**D**). **Z**^amp^(*ω*) visualizes the importance of the connections for the peak amplitude and **Z**^freq^(*ω*) the importance for the peak frequency. Same display as in Fig 5C.

From Fig 4 we identify four modes that potentially contribute to the generation of the high-*γ* peak. Evaluating the sensitivity measure for these modes at their respective peak frequencies reveals each mode being shaped by the self-coupling of one inhibitory population (Fig 6). This mechanism has been termed ING [20] and the peak frequency of the modes and the resulting peak in the spectrum depends on the delay of the synapses, the refractory time and the decay time of the IPSPs [17]. Inserting the time constants used in the microcircuit model into Eq (15) of [17], predicts an oscillation frequency of 288 Hz corresponding to the high-*γ* oscillations observed in the simulations. The rapidity of the high-*γ* oscillation in the model is hence explained by the choice of small time constants for the IPSCs (*τ*_syn_ = 0.5 ms). Larger synaptic time constants yield an ING peak of lower frequency, for example 127 Hz, 97 Hz and 80 Hz for time constants of 2 ms, 3 ms and 4 ms, respectively. In the original microcircuit model the synaptic time constants are chosen to be small and equal for all neurons to investigate the contributions of the connectivity to the emergent dynamics. The formalism developed in [54] and [50] delivers good predictions for the stationary firing rate and transfer function of the populations for synaptic time constants in the range of a few milliseconds and therefore provides the basis of a successful application of the mean-field and linear response theory. For larger time constants the analytically predicted transfer function can still serve as an approximation to predict the tendencies of the population rate spectra. When the synaptic time constant exceeds the membrane time constant (*τ*_syn_ > *τ*_*m*_) an adiabatic approximation [55] is applicable. The dominant mode determining the high-*γ* oscillation of the full circuit originates in the self-coupling of 4I (Fig 6A). The sign of the entries in **Z**^amp^ and **Z**^freq^ reveals that an increase in the high-*γ* oscillation, by alterations of the connectivity, goes along with an increase in the oscillation frequency and vice versa. Adjustments of the I-I-loop within layer 4 has an opposite effect than alterations of the I-I-loops within other layers. It turns out that the eigenvalue corresponding to the dominant mode has a real part which is slightly larger than one. Weakening the connections in the 4I-4I-loop stabilizes the circuit dynamics. Once the trajectory is shifted past one, the sensitivity measures takes the opposite sign and predicts decreased high-*γ* oscillations when connections from 4I to 4I are removed. This shift of the eigenvalue from real parts larger than one to real parts smaller than one describes the transition of network dynamics from the SI to the AI regime [introduced in 12]. Interestingly, in the stabilized circuit (see “*Stability of the dynamical modes*”), the alterations of connections from 4I to 4I has opposing effects on the amplitude and frequency of the low-*γ* (Fig 5C) and the high-*γ* oscillation.

### Slow rate fluctuations

**Fig 7:**
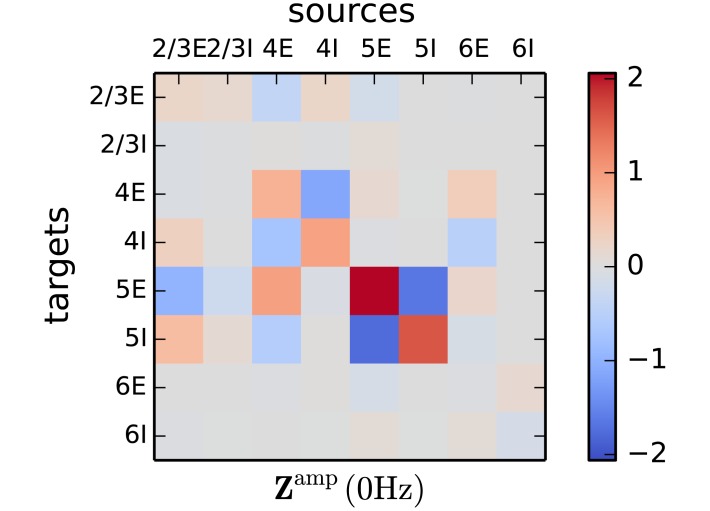
Connections relevant for low frequency oscillations. The matrix elements show the sensitivity of the peak amplitude of low frequency fluctuations on the individual connections. Same display as in Fig 5C.

**Fig 8:**
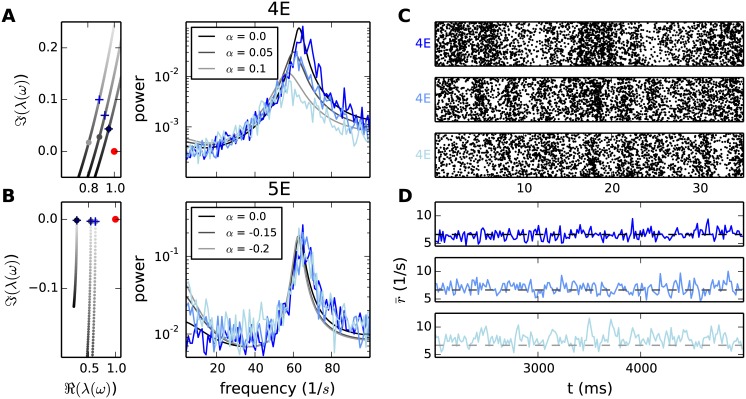
Targeted alteration of the low-*γ* peak and slow fluctuations. **A** Increased number of connections from 4I to 4I in the microcircuit by fraction *α* of 5% and 10% (legend). Left panel: section of the eigenvalue trajectories (50 Hz-70 Hz) associated with the low-*γ* peak of the two altered and the original circuit. The positions of the large gray dots denote the eigenvalue at the peak frequency of the original data set and their shading the value of *α*. The crosses mark the position where the new eigenvalue trajectory passes closest to one. Since the crosses appear before the dots, the new peak is shifted to lower frequencies. The red dot denotes the critical value one. Right panel: spectra of the simulated circuits (blue, shading as for gray in legend) and the analytical predictions (gray). **B** Removing 15% and 20% (legend) of the connections from 5E to 5I in the microcircuit. Left panel: section of the eigenvalue trajectories (0 Hz-10 Hz) associated with the slow rate fluctuations of the two altered and the original circuit. Same display as in A. Right panel: spectra of the simulated circuits (blue) and the analytical predictions (gray). **C** All spike times of the neurons in population 4E occurring in a time segment of 35 ms, for the three parameter regimes introduced in A (*α* increases from top to bottom). **D** Instantaneous firing rate of population 5E (binning window: 15 ms), in a time segment of 3 s, for the three parameter regimes introduced in B (*α* decreases from top to bottom). The dashed lines show the theoretical predictions of the stationary rates.

Since the sensitivity measure analyzes the eigenvalues of the effective connectivity matrix, it sheds light on the static properties of the circuit when evaluated at zero frequency (Fig 7). The eigenvalue with the largest real part determines the stability of the circuit. At the same time, the measure evaluated at zero frequency reveals the connections shaping low frequency fluctuations. These two statements describe the same phenomenon, since a circuit near an instability exhibits slowly decaying modes when perturbed in the direction of the eigenmode corresponding to the eigenvalue with the largest real part. Technically, there is a peak at zero frequency, but in practice the power in a wide range of low frequencies is elevated, as visible in the spectra in Fig 8B and in the corresponding traces of instantaneous firing rates in Fig 8D.

The largest entries of the sensitivity measure evaluated at zero frequency correspond to connections within layer 5. This finding is in agreement with experimental literature [38, 39], where the onset of slow fluctuations was observed to be initiated in layer 5, as recorded in the sensory-motor areas of the mouse and area V1 of the cat. In contrast to the low- and high-*γ* oscillations, the slow oscillations are independent of the delay and time course of the neuronal responses. Thus, the amplitude of the slow fluctuations depends solely on the anatomical connections of the circuit and the slope of the f-I curve of the neurons. The indegree matrix (Fig 1C) shows that the number of connections from 5E to 5I is low compared to other indegrees in layer 5. The reduced excitatory input to 5I results in lower rates of the inhibitory neurons relaying less inhibition back to 5E. The comparably stronger E-E-loop is driven towards a rate instability, exhibiting slowly decaying modes of the population rate, which appear as low frequency components in the spectrum. In agreement with the previous considerations, the slow oscillations become stronger if the self-coupling of the populations in layer 5 is strengthened and weaker if the cross-coupling is increased Fig 7. Further relevant connections are located within layer 4 and starting in population 2/3E and 4E projecting onto layer 5. The measure predicts that strengthening connections from 2/3E to 5E reduces slow oscillations.

### Influence of single connections on the spectra

We now exploit the sensitivity measure to predict changes in the spectra in different frequency ranges when individual connections are altered. The predictions are validated by simulations of the microcircuit with perturbed indegrees. According to the sensitivity measure shown in Fig 5C, increasing the self-coupling of population 4I should lower the amplitude of the low-*γ* peak and decrease the frequency. Simulations confirming these predictions are shown in Fig 8A. Since we are interested in the contribution of the connection to fluctuations of the activity, we fix the dynamical state of the populations by simultaneously decreasing the external input to 4I. The left panel demonstrates the shift of the eigenvalue trajectory when altering the connectivity. Since the connection from 4I to 4I strongly influences the dynamics at 64 Hz (Fig 5), while having small impact on the low frequency spectrum (Fig 7), the spectrum produced by the altered circuitry deviates from the original one only at frequencies around the low-*γ* peak. Simulating the microcircuit for increased self-coupling of population 4I confirms the theoretical predictions. Note that reducing the number of synapses from one connection in the microcircuit by as little as 10% can cause an attenuation of the peak amplitude to 7% of its original value and a frequency shift of 11 Hz. The reduction of the oscillation is also visible in the spiking activity. Simultaneously inspecting all spike times of the neurons in population 4E (Fig 8C top), we observe three population burst for the circuit with the original connectivity. The populations bursts become less prominent (Fig 8C, middle and bottom) when the number of connections from 4I to 4I is increased, an observation which is in agreement with the predicted population rate spectra shown in Fig 8A. Given that layer 4 is the input layer, we show here that the spectrum exhibited by the circuit is highly sensitive to variations within layer 4, which could originate either from within the circuit or from external drive.

Starting from the hypothesis that slow rate fluctuations are controlled within layer 5, suggested by the sensitivity measure (Fig 7), we perturb the indegree from 5E to 5I. The right panel in Fig 8B shows the expected increase of the peak at low frequencies in the spectrum of 5E for fewer connections from 5E to 5I. The predictions match the simulation results. The low frequency oscillations are reflected as slow rate fluctuations in the instantaneous firing rates (Fig 8D). While the stationary firing rate of population 5E is almost not affected by perturbation of the connectivity (compare the positions of the dashed lines in the three panels of Fig 8D), the amplitude of the rate fluctuations increases visibly.

The enhanced peak amplitude of the spectrum is explained by the onset of the corresponding eigenvalue trajectory being shifted towards one (left panel, Fig 8B). In agreement with the prediction of the sensitivity measure, the spectrum for frequencies above 20 Hz is unaffected by alterations of the connectivity in layer 5. Thus we conclude that layer 5 is capable of locally eliciting slow rate fluctuations while leaving the properties of the full circuit at high frequencies unimpaired.

### Anatomical origin of low-*γ* oscillations

The preceding sections investigate how the sensitivity measure predicts the influence of individual connections on the spectrum. Next we apply these insights to uncover the minimal circuitry generating the 64 Hz oscillation. The sub-circuit is obtained by starting from an unconnected circuit, i.e. missing input from other populations is compensated by Poisson spike trains with the same mean and variance. In this setup the populations display the same stationary firing rates as in the original network, but the correlations on the population level are negligible, resulting in a flat population rate spectrum. The empty connectivity matrix is then successively filled with the connections that have largest entries in **Z**^amp^(64 Hz) and **Z**^freq^(64 Hz), while ensuring stability of the resulting system (instabilities can arise when adding an excitatory connection without an inhibitory counterpart). We continue this procedure in decreasing order of sensitivity until the peak frequency of the original spectrum is approximately restored. It turns out that the five largest entries of **Z**^amp^ and the eight largest entries of **Z**^freq^ suffice to reproduce at least 95% of the peak frequency and 83% of the logarithmic peak amplitude in all populations contributing to the circuit. Fig 8A visualizes the resulting connectivity along with simulation results of the reduced circuit and the analytical prediction of the spectra for the original and the reduced circuit.

**Fig 9:**
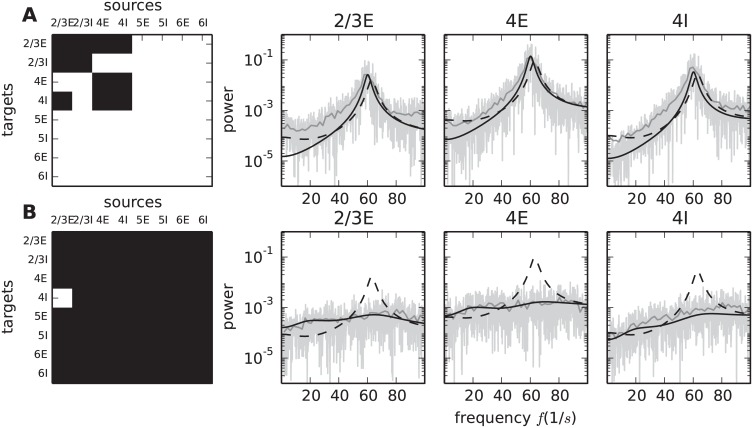
Minimal circuit for low-*γ* oscillations. **A** Left: Connectivity of the minimal circuit generating the 64 Hz oscillation. The circuit is composed of the connections corresponding to the five largest matrix elements of **Z**^amp^(64 Hz) and the eight largest elements of **Z**^freq^(64 Hz) forming the black mask; all other elements are set to zero (white). Right: Rate spectra for three populations (graph titles) obtained by direct simulation of the reduced circuit (gray curves, methods as in Fig 1E) with the input from missing connections replaced by Poisson input, in comparison to the analytical prediction of the spectra in the full (black dashed curves) and the reduced circuit (black solid curves). **B** Left: Connectivity of the original circuit with only the connection from 2/3E to 4I taken out; black mask indicates that only this matrix element is set to zero (white). Right: Same display as in panel A for the original circuit with the connection from 2/3E to 4I replaced by Poisson input corresponding to the rate of 4I. The dashed black curve is the analytical prediction for the original circuit (same curve as in A), the solid black curve is the prediction for the modified circuit.

Confirming our previous conclusions, the minimal circuit is located in a sub-system composed of layers 2/3 and 4. The blocks along the diagonal in Fig 9A show that all connections within layers 2/3 and 4 contribute to the minimal circuit. Additional connections start in the populations of layer 4 and terminate in population 2/3E. The loop is closed by the projection from population 2/3E to the inhibitory population in layer 4, revealing the special role of this connection in ensuring the recurrence of the oscillation-generating circuit. Testing this hypothesis, we simulate the circuit with the original connectivity, leaving out the connection from 2/3E to 4I. As predicted the peak vanishes entirely (Fig 9B).

In summary, these considerations show that given the dynamical state of the populations in the microcircuit, the circuit depicted in Fig 9A is shaping the spectrum around 64 Hz. However, the spectrum generated by the sub-circuit in isolation, i.e. without the substitution of the input of other populations by Poisson spike trains, would potentially be different. Here the advantage of the two-step reduction in the derivation of the theoretical framework becomes apparent. Performing the diffusion approximation the firing rates and response properties of the populations are established and can be verified by experimental data. The analysis of the dynamical contributions of the individual connections or sub-circuits can then be conducted after having fixed these quantities.

## Discussion

In the present work we investigate the oscillations generated in a spiking microcircuit model [34], which integrates knowledge from more than 50 anatomical and physiological studies. We show that this level of abstraction suffices to reproduce experimentally observed laminar specific oscillation patterns, such as the generation of high frequency oscillations in the *γ* range in upper layers [40, 56, 57, 21] and lower frequencies in deeper layers [38, 35, 36, 39]. In particular, we derive a sensitivity measure, starting from a theoretical description of the underlying spiking neuron model by a combination of mean-field and linear response theory. The measure yields a state dependent dynamic connectivity map from which we extract the minimal circuit that shapes the oscillations. Different dynamical states of the network, elicited by changes in the input or noise induced switching due to multiple underlying fixed points of the stationary dynamics therefore exhibit different values of the sensitivity measure, i.e. the importance of individual connections for the oscillations generally varies for different dynamic states. The presented sequence of theoretical arguments leads to a simple visualization technique providing an intuitive understanding of the stability and oscillatory behavior of the circuit when changing connection parameters.

### Main findings in the microcircuit model

The sensitivity measure reveals that the peak in the low-*γ* range is generated by a sub-circuit consisting of layer 2/3, layer 4 and the connections from layer 4 to 2/3E and from 2/3E to 4I. This finding is in agreement with experimental literature locating *γ* oscillations in the upper layers. Furthermore, we identify the feedback connection from 2/3E to 4I and the feed-forward connections from layer 4 to layer 2/3 as crucial for the amplitude of the peak. The oscillation generated by the cooperation of the two upper layers is of lower frequency than the oscillation produced by the layers in isolation. A hint on layers 2/3 and 4 teaming up to generate a low frequency *γ* peak has been found in Ainsworth et al. [58]. The frequency of the peak is predominantly determined by connections within the input layer 4. This implies that excitation of the column will be reflected in a frequency shift of the *γ* peak, which results from an alteration of the dynamical state of the populations and therefore of the effective connectivity. The variability of the generated frequency caused by inputs to layer 4 has been demonstrated experimentally [18, 59]. The collective oscillations could also be shaped by alterations of the synaptic efficacies between layers 2/3 and 4 (e.g. by short term plasticity). Further experimental studies need to probe the influence of perturbations in weight and number of synapses on the amplitude and frequency of *γ* peaks in the population rate spectra. The sensitivity measure can be utilized to verify the parameters used in the model and to reveal shortcomings of the theoretical description, which potentially arise from the assumptions of simplified neuron-models and negligible auto-correlations.

High-*γ* peaks are found to be generated in the I-I-loops of each layer, with the loop in layer 4 dominating the spectra. This mechanism, termed ING, has been analyzed previously [20] and experimentally located in upper layers. In the microcircuit, the second largest contribution arises from the I-I-coupling in layer 6; we hence propose to target this layer experimentally to test this hypothesis.

Connections determining slow rate fluctuations and the stability of the circuit are identified by the sensitivity measure at zero frequency. The measure shows that connections within layer 5 as well as the connections from population 2/3E and 4E to layer 5 are crucial. We conclude that there are too few connections from 5E to 5I to counteract the rate fluctuations which accumulate due to the amplification within the strong 5E-5E loop. Our findings are in good agreement with experimental results demonstrating the initiation of slow frequency oscillations in layer 5, as well as the stronger amplification of low frequency oscillations in response to a stimulus applied to layer 5 than to a stimulation of layer 2/3 [38]. Given the dynamical state of 5E, the circuit is stabilized when removing connections from 2/3E to 5E, resulting in a decrease of slow rate fluctuations. In contrast, an impairment of the connections from 4E to 5E has the effect of strengthening the self-amplification of fluctuations and therefore strengthens slow oscillations. With the emerging optogenetic toolbox it may be possible to experimentally test these two predictions in the future.

Our analysis suggests a refinement of the parameters of the microcircuit model, which are so far deduced from direct measurements of anatomical and physiological connectivity alone [34]. Experimental studies show that the amplitude of *γ* oscillations depends on the stimulus strength [60], suggesting that the current microcircuit model captures the cortical tissue in a semi-stimulated regime. Lowering the external input to the excitatory neurons in layer 4 decreases the low-*γ* power in the idle state, which in addition sensitizes population 4E to evoke *γ* oscillations when stimulated.

### Contributions of synaptic delays

Synaptic delays do not influence the stationary state of the network, characterized by the time-averaged firing rates of all populations, but crucially shape the fluctuations around this stationary set point. We provide an intuitive understanding of the influence of delays on oscillations with parametric plots of the eigenvalues of the activity modes determining the spectra of the circuit. Small delays cause fast oscillations, while long delays support slow ones. Larger delays move the network towards the regime of sustained oscillations, which is counteracted by heterogeneity in the delays. The frequency of the oscillation is highly sensitive to the delays, but the static properties of the circuit, which depend on the dynamic state of the neurons and the anatomical connectivity, determine whether a network displays fast or extremely slow oscillations.

### Applicability of the sensitivity measure

The newly derived sensitivity measure determines crucial connections for the frequency and amplitude of population rate oscillations. Since its applicability is not constrained to the analysis of indegrees, it permits a systematic investigation of complicated networks with respect to parameters such as the synaptic delay, connection weight, or excitation-inhibition balance. In these pages we exemplified its use by the analysis of a particular model, but it can in principle be utilized to identify dynamically relevant circuits embedded in any high-dimensional network. Our work thus extends existing methods analyzing single- or two-population network models to more intricate structures. The significance of the identified connections is validated by demonstrating how small changes in the number of synapses can have a large impact on the spectra of all populations.

The formalism requires the neurons to work in a regime where the activity fluctuations of the inputs are summed linearly on the considered time scale. Simulations of networks of LIF-model neurons confirm the validity of the linear approximation. Experimental evidence supports the existence of cortical networks operating in this regime [61, 62, 63, 64]. Since the sensitivity measure can be applied to any network whose dynamics can be approximated by a linear rate model, the applicability goes beyond circuits composed of LIF-model neurons. For example, responses of modified IF models have been shown to approximate neural responses in vivo [65]. Several studies treat the stationary and dynamical properties of these models in the linear regime (see [66] for EIF and [67] for QIF). Grabska-Barwinska et al. [68] emphasize that theoretical predictions for networks composed of QIF neurons in the asynchronous regime, by trend, also hold in networks operating in a more synchronized regime, in which individual neurons are exposed to larger input fluctuations. For neuron models with conductance-based synapses a reduction to effective current based synapses exists [69, 70] and therefore enables the usage of the theoretical framework developed in [13, 12]. Furthermore, networks of current and conductance based model neurons have been pointed out to be qualitatively comparable (see section 3.5.3. in [65]). Alternatively the measure can be fed with experimentally obtained firing rates and transfer functions [71, 61, 72] of neuronal populations to analyze the underlying circuits generating the oscillations.

The proposed method also finds application in systems where the non-linearities affect the dynamics on a slower time scale than the considered oscillation. Such non-linearities can be taken into account by reevaluating the measure for different mean-inputs corresponding to different phases of the slow input fluctuations. Employing the measure in the described iterative fashion results in a phase-dependent identification of relevant connections for the generation of the fast rhythm and thus sheds light on the anatomical origin of phase-amplitude coupling [reviewed in 21].

The method can also be exploited in reverse to engineer circuits with a desired oscillatory behavior in a top-down fashion.

The results presented here lead to clear interpretations of experimental data on network activity and to new hypotheses. It should be noted, however, that the model of the microcircuit represents an early draft and was purposefully designed by its authors as a minimal model with respect to the number of populations and the heterogeneity in the neuronal dynamics. Therefore, failure in the reproduction of certain phenomena found in nature or in the confirmation of a hypothesis should not be attributed to the mathematical method developed here, but to shortcomings of the investigated model. The method is applicable to any update of the original model as structural data and single neuron properties become more refined, given that the assumptions underlying the mean-field and linear response theory are still met. One potential extension is the subdivision of the inhibitory neurons into multiple populations representing different types of inter-neurons, with connection probabilities that yield specific connectivity motifs, as recently reported in [73].

The sensitivity measure uncovers the contribution of the laminar structure to the population rate spectra and produces predictions which can be tested experimentally by comparing the spectra generated by different species or brain areas with distinct laminar structures.

In summary the current work introduces a method which elucidates the relation between anatomy and dynamical observables of layered cortical networks. Even though a specific model is used to exemplify the method and to derive concrete predictions, the novel method provides a general framework for the systematic integration of the anatomical and physiological data progressively becoming available into ever more consistent models of cortical circuitry.

## Methods

### Spiking model of a microcircuit

While analyzing the oscillatory properties of the microcircuit model in this work it turned out that the model with its original parameters [as specified in Table 5 of 34] is in a dynamical regime very close to the onset of sustained population oscillations, resulting in spectra with distinct frequency peaks. We stabilized the circuit by removing 15% of the connections from 4I to 4E and increasing the standard deviation of the delay distribution of all connections to 1 ms. To keep the rates fixed we compensate for the lack of inhibitory input to 4E by removing 19% of the external excitatory input.

All simulations were carried out using the simulation software NEST [74]. The source code describing the cortical microcircuit is included in the examples within the release package of NEST as of version 2.4.

The population rate spectra from simulations are computed by extracting the spike trains of all neurons in one population from a simulation of 10 s duration and subsequently applying the Fast Fourier Transform (FFT) algorithm using a binning of 1 ms. In addition we provide spectra obtained by averaging over 500 ms windows.

### Fluctuation dynamics

We here use the term “mean-field theory” for the first step of our analysis, i.e. the equation determining the time-averaged activity characterized by the firing rates of the neurons. This notion, to our knowledge, has its origin in the literature on disordered systems [75, 76, 77] and entered the neuroscience literature by the works of Amit et al. [46] for spiking model neurons, Sompolinsky et al. [78] for non-linear rate models and van Vreeswijk et al. [79] for binary model neurons. Note that these theories include synaptic fluctuations. In contrast, mean-field theory in its original meaning is applied to systems without disorder, where it follows from the lowest order saddle point approximation in the local order parameter (see e.g. [80], Chapter 4.3, Ferromagnetic transition for classical spins), which neglects fluctuations altogether.

In the second step of our analysis, we employ linear response theory to characterize the dynamical properties of the populations by a transfer function [13, 81, 50]. On the basis of this ingredient, we utilize the finding that a linear rate model with output noise [49] captures the dynamics of circuits composed of LIF-model neurons in the asynchronous irregular regime. The term “rate model” is used in its general sense, as a set of coupled stochastic differential or convolution equations of time-dependent signals. Therefore the observed population-averaged spiking activity *y*_*i*_(*t*) of the *i*-th population can be interpreted as the fluctuating time density of spike emission *r*_*i*_(*t*) of the neurons with an additive noise component *x*_*i*_(*t*) obeying

$$\begin{array}{c} y_{i}\left(t\right)=r_{i}\left(t\right)+x_{i}\left(t\right),\;\left\langle x_{i}\left(t\right)\right\rangle =0\\ \left\langle x_{i}\left(s\right)x_{j}\left(t\right)\right\rangle =\delta_{ij}\delta \left(s-t\right)\frac{{\bar{r}}_{i}}{M_{i}},\;i,j\in \left\{E,I\right\}. \end{array}$$ (10)

The white noise effectively describes the fluctuations caused by the spiking realization of the point process. Here ${\bar{r}}_{i}$ denotes the average rate of the population with size *M*_*i*_ and the last line shows that the noise produced by different populations is uncorrelated. Correlations between the populations are induced by the connectivity of the network of populations. The rate *y*_*i*_(*t*) describes a signal which fluctuates around the offset *r*_*i*_(*t*). The amplitude of these fluctuations is infinite in the precisely defined sense of a white noise [82]. The necessity for this additive white noise arises from demanding the equivalence between the original spiking signal and its stochastic counterpart *y*_*i*_(*t*) Eq (10) on the level of their pairwise statistics: the noise for a rate signal corresponding to a single spike train has to be chosen such that the autocorrelations of the two signals agree. In this case, the white noise generates a Dirac *δ* peak weighted by the firing rate. The additional factor 1/*M*_*i*_ in Eq (10) arises from the uncorrelated superposition of *M*_*i*_ such signals [for the formal derivation cf. 49, esp. Section 4]. Even though the white noise formally has infinite variance, all observable quantities, namely averages over short time intervals, exhibit a finite variance corresponding to that of a Poisson process. In other words, binning a sufficiently long time series *y*(*t*) with bin size Δ*t*, the variables $\tilde{y}\left(t\right)=\frac{1}{\Delta t}\int_{t}^{t+\Delta t}y\left(t^{\prime }\right)\;dt^{\prime }$ (describing the observed fluctuating spike density in each bin) are characterized by a distribution with mean *r*(*t*) and variance $\frac{\bar{r}}{M}\frac{1}{\Delta t}$.

In this work the validity of the linear approximation is tested by simulations of networks of LIF-model neurons, expressing a non-linearity by their hard threshold on the membrane potential. The description suffices since the network-generated noisy activity effectively linearizes the response of the neurons. This is a fundamental property of non-linear systems subject to noisy inputs, often studied in the context of stochastic resonance in biology [83, 84, 85] and reviewed in [86].

In signal processing, the impulse response characterizes the output of a system after the application of a short external input [51]. The time fluctuations of the population rates are obtained by integration over the history of all incoming impulses convolved by the impulse response *H*_*ij*_(*t*)

$$r_{i}\left(t\right)=\int_{-\infty }^{t}\sum_{j=1}^{N}M_{ij}^{A}H_{ij}\left(t-s\right)\left(r_{j}\left(s-d_{ij}\right)+x_{j}\left(s-d_{ij}\right)\right)\;ds,$$ (11)

where *d*_*ij*_ denotes the delay of the connection from population *j* to *i*. The impulse response *H*_*ij*_(*t*) of a population of LIF-neurons is obtained by applying linear response theory to the corresponding Fokker-Planck Eq (13). We here use the recently derived extension incorporating exponentially decaying synaptic currents [50, Eq (30)]. The effective connectivity matrix **M**(*t*) with elements

$$M_{ij}\left(t\right)=M_{ij}^{A}H_{ij}\left(t\right)$$ (12)

summarizes the rate response of population *i* to an impulse sent from population *j*. This matrix has two contributions. The first part, termed the anatomical connectivity $M_{ij}^{A}$, determines the size of the incoming input. The anatomical connectivity matrix is element-wise composed of the indegree matrix **K** and the weight matrix **W**

$$M_{ij}^{A}=K_{ij}W_{ij},\;W_{ij}=\left\{\begin{array}{cc} J_{E} & \text{if}\;j\in E\\ J_{I} & \text{if}\;j\in I \end{array}\right..$$ (13)

Here *K*_*ij*_ describes the number of incoming connections from population *j* to population *i* and *W*_*ij*_ their respective weight. The second part describes the time course of the rate response *H*_*ij*_(*t*). The substitution *s* → *s* + *d* when integrating Eq (11) permits the absorption of the time delay into the effective connectivity matrix **M**_d_(*t*) = **M**(*t* − *d*). Transforming Eq (11) into Fourier space yields

$$\begin{array}{c} R\left(\omega \right)={\tilde{M}}_{d}\left(\omega \right)\left(R\left(\omega \right)+X\left(\omega \right)\right)\\ \Rightarrow R\left(\omega \right)={\left({\tilde{M}}_{d}^{-1}\left(\omega \right)-I\right)}^{-1}X\left(\omega \right) \end{array}$$ (14)

with ${\tilde{M}}_{d,ij}\left(\omega \right)={\tilde{M}}_{ij}\left(\omega \right)e^{-i\omega d_{ij}}$. Since the delays are Gaussian distributed we need to average over all possible realizations of the delays. This averaging can formally be done by weighting the contributions involving the delays with the probability density function *f*(*y*) describing the delay distribution $e^{-i\omega d_{ij}}\to \int_{-\infty }^{\infty }e^{-i\omega y}f\left(y\right)\;dy$. Here the probability function is given by a renormalized Gaussian distribution truncated at zero (since the delays are positive), yielding the effective connectivity

$$\begin{array}{ccc} {\tilde{M}}_{d,ij}\left(\omega \right) & = & \frac{{\tilde{M}}_{ij}\left(\omega \right)}{\sqrt{2\pi }\sigma_{d_{ij}}\left(1-\Phi \left(\frac{-d_{ij}}{\sigma_{d_{ij}}}\right)\right)}\int_{0}^{\infty }dy\;e^{-i\omega y}e^{-\frac{{\left(y-d_{ij}\right)}^{2}}{2\sigma_{d_{ij}}^{2}}},\\ & = & \frac{1-\Phi \left(\frac{-d_{ij}+i\omega \sigma_{d_{ij}}^{2}}{\sigma_{d_{ij}}}\right)}{1-\Phi \left(\frac{-d_{ij}}{\sigma_{d_{ij}}}\right)}{\tilde{M}}_{ij}\left(\omega \right)e^{-i\omega d_{ij}}e^{-\frac{\sigma_{d_{ij}}^{2}\omega^{2}}{2}} \end{array}$$ (15)

with $\sigma_{d_{ij}}$ being the standard deviation of the delay from population *j* to population *i*, *d*_*ij*_ the average delay, and

$$\Phi \left(x\right)=\frac{1}{2}\left(1+\text{erf}\;\left(\frac{x}{\sqrt{2}}\right)\right),$$

with the error function erf(*x*). The integration can be performed for any probability density function. Therefore the formalism generalizes to models incorporating delay heterogeneities with other statistics than a Gaussian distribution. The activity composed of the output rate and the additional noise is thus given by

Y(ω)=R(ω)+X(ω)=P(ω)X(ω), (16)

where we define $P\left(\omega \right)={\left(I-{\tilde{M}}_{d}\left(\omega \right)\right)}^{-1}$ as the propagator determining how the noise is mapped via the network onto the observable activity **Y**. The cross-correlations between the activities are given by

$$C\left(\omega \right)=\left\langle Y\left(\omega \right)Y^{T}\left(-\omega \right)\right\rangle =P\left(\omega \right)DP^{T}\left(-\omega \right),$$ (17)

where **D** = 〈**X**(*ω*)**X**^*T*^(− *ω*)〉 is the diagonal matrix of correlations between the effective noise sources **X**, which represent the spiking realization of the neuronal signals. Due to the initial independence of the neurons Eq (10), the correlation matrix has diagonal form with the elements defined by the average firing rate of the neurons and the population size ($D_{ii}={\bar{r}}_{i}/M_{i}$). The stationary firing rates of LIF model neurons supplied with colored noise is derived in Fourcaud et al. [47]. The spectrum of the *i*-th population can be directly read off the diagonal of the cross-correlation

$$C_{ii}\left(\omega \right)={\left\langle Y\left(\omega \right)Y^{T}\left(-\omega \right)\right\rangle }_{ii}.$$ (18)

### Frequency dependent eigenmode decomposition

In Fourier space the effective connectivity matrix is a function of frequency *ω*. For every frequency the matrix can be decomposed, resulting in *N* = 8 eigenvalues with the corresponding left and right eigenvectors

$$\begin{array}{c} \tilde{M}\left(\omega \right)u_{i}\left(\omega \right)=\lambda_{i}\left(\omega \right)u_{i}\left(\omega \right)\\ v_{i}^{T}\left(\omega \right)\tilde{M}\left(\omega \right)=\lambda_{i}\left(\omega \right)v_{i}^{T}\left(\omega \right). \end{array}$$ (19)

The eigenvectors are normalized such that the product of the left and right eigenvector equals one. The propagator shares its eigenvectors with the effective connectivity matrix and the eigenvalues are given by

$$P\left(\omega \right)u_{i}\left(\omega \right)=\frac{1}{1-\lambda_{i}\left(\omega \right)}u_{i}\left(\omega \right).$$ (20)

The noise can be expressed in the new basis as

$$X\left(\omega \right)=\sum_{i}\alpha_{i}\left(\omega \right)u_{i}\left(\omega \right),\;\alpha_{i}\left(\omega \right)=v_{i}^{T}\left(\omega \right)X\left(\omega \right).$$ (21)

Hence the cross-correlations in the new basis take the form

$$C\left(\omega \right)=\sum_{i,j=1}^{N}\underset{≕\beta_{ij}\left(\omega \right)}{\underset{︸}{\frac{\alpha_{i}\left(\omega \right)\alpha_{j}^{*}\left(\omega \right)}{\left(1-\lambda_{i}\left(\omega \right)\right)\left(1-\lambda_{j}^{*}\left(\omega \right)\right)}}}\underset{≕T_{ij}\left(\omega \right)}{\underset{︸}{u_{i}\left(\omega \right)u_{j}^{*T}\left(\omega \right)}}.$$ (22)

Here **T**_*ij*_(*ω*) is the matrix given by the outer product of the eigenvectors of the *i*-th and *j*-th mode evaluated at frequency *ω*, where we employed $u_{i}\left(-\omega \right)=u_{i}^{*}\left(\omega \right)$. This relation holds since the impulse response *H*_*i*_(*t*) entering the effective connectivity matrix is real valued in the time domain.

When one eigenvalue approaches unity at frequency *ω*_0_ (λ_*c*_(*ω*_0_) ≈ 1), the spectrum at this frequency is dominated by the contribution of the critical mode *c* and we can approximate the spectrum visible in the *k*-th population by

$$C_{kk}\left(\omega_{0}\right)\approx |\frac{\alpha_{c}\left(\omega_{0}\right)}{1-\lambda_{c}\left(\omega_{0}\right)}{|}^{2}u_{c,k}\left(\omega_{0}\right)u_{c,k}^{*}\left(\omega_{0}\right)=\beta_{cc}\left(\omega_{0}\right)\;T_{cc,k}\left(\omega_{0}\right).$$ (23)

### Frequency independent eigenmode decomposition

In a simplified circuit with all populations having the same transfer function *H*(*ω*) the eigenvalue decomposition of the effective connectivity matrix reads

$$\tilde{M}\left(\omega \right)=H\left(\omega \right)\sum_{i=1}^{N}\lambda_{i}^{A}u_{i}^{A}v_{i}^{A,T}.$$ (24)

Here $\lambda_{i}^{A}$ is the *i*-th eigenvalue of the anatomical connectivity matrix and $u_{i}^{A}$ and $v_{i}^{A}$ are the associated right and left eigenvectors, respectively. The propagator matrix Eq (20), mapping the noise of the system to the rate, is determined by the effective connectivity matrix and thus has the same eigenvectors and the eigenvalues $1/(1-H\left(\omega \right)\lambda_{i}^{A})$. Mapping the rate vector **R**(*ω*) into the coordinate system spanned by the right and left eigenvectors of the anatomical connectivity matrix ($u_{i}^{A},\;v_{i}^{A,T}$), the rates of the initial populations $R_{i}\left(\omega \right)=e_{i}^{T}R\left(\omega \right)$ (where $e_{i}$ is the unit vector being one at position *i* and zero everywhere else) are converted to the dynamic modes $v_{i}^{A,T}R\left(\omega \right)$. Fig 3A shows a scheme of the coordinate transformation. The activity of the *i*-th mode is fed back solely to itself with the connection weight $\lambda_{i}^{A}v_{i}^{A,T}u_{i}^{A}$ and filtered by the transfer function *H*(*ω*).

By expressing the ongoing spiking activity propagating through the system Eq (21) as a linear combination of the eigenmodes, the total activity is described by the sum of the activity of decoupled modes. The diagonal elements of the cross-correlation matrix describing the spectrum of the populations can be expressed in the new basis

$$C_{kk}\left(\omega \right)=\sum_{i,j=1}^{N}\underset{≕\beta_{ij}^{A}\left(\omega \right)}{\underset{︸}{\frac{\alpha_{i}\left(\omega \right)\alpha_{j}^{*}\left(\omega \right)}{\left(1-H\left(\omega \right)\lambda_{i}^{A}\right)\left(1-H^{*}\left(\omega \right)\lambda_{j}^{A*}\right)}}}\underset{≕T_{ij,k}^{A}}{\underset{︸}{u_{i,k}^{A}u_{j,k}^{A*}}},\;k\in 1,..,N,$$ (25)

with $\alpha_{i}\left(\omega \right)=v_{i}^{A,T}X\left(\omega \right)$ being the projection of the noise into the new coordinate system. The contribution of one mode dominates if $H\left(\omega_{0}\right)\lambda_{c}^{A}\approx 1$ and we can approximate the spectrum at *ω*_0_ with

$$C_{kk}\left(\omega_{0}\right)\approx |\frac{\alpha_{c}\left(\omega_{0}\right)}{1-H\left(\omega_{0}\right)\lambda_{c}^{A}}{|}^{2}u_{c,k}^{A}u_{c,k}^{A*}=\beta_{cc}^{A}\left(\omega_{0}\right)\;T_{cc,k}^{A}.$$ (26)

### Dynamical modes

This section devises a method to break a circuit down into smaller independent circuits each describing distinct characteristics of the spectrum by means of eigenvalue decomposition of the effective connectivity matrix. The activity $R_{k}\left(\omega \right)=e_{k}^{T}R\left(\omega \right)$ of population *k* is given by

$$R_{k}\left(\omega \right)=\sum_{l=1}^{N}{\tilde{M}}_{d,kl}\left(\omega \right)\left(R_{l}\left(\omega \right)+X_{l}\left(\omega \right)\right)=H_{k}\left(\omega \right)\sum_{l=1}^{N}M_{kl}^{A}\left(R_{l}\left(\omega \right)+X_{l}\left(\omega \right)\right).$$ (27)

and illustrated in the top of Fig 3A.

We now consider a simplified circuit where all populations have the same transfer functions. Here, the anatomical and dynamical part of the effective connectivity can be treated separately

$${\tilde{M}}_{ij}\left(\omega \right)=H\left(\omega \right)\;M_{ij}^{A}.$$ (28)

The anatomical part $M_{ij}^{A}$ can be split into eight modes using eigenvalue decomposition Eq (24) yielding the activity of one eigenmode ${\tilde{R}}_{k}\left(\omega \right)=v_{k}^{A,T}R\left(\omega \right)$

$$\begin{array}{ccc} & {\tilde{R}}_{k}\left(\omega \right) & =v_{k}^{A,T}\sum_{i=1}^{N}\lambda_{i}^{A}H\left(\omega \right)u_{i}^{A}v_{i}^{A,T}\left(R\left(\omega \right)+X\left(\omega \right)\right)\\ & & =\lambda_{k}^{A}H\left(\omega \right)v_{k}^{A,T}\left(R\left(\omega \right)+X\left(\omega \right)\right)=\lambda_{k}^{A}H\left(\omega \right)\left({\tilde{R}}_{k}\left(\omega \right)+{\tilde{X}}_{k}\left(\omega \right)\right), \end{array}$$

as visualized in the bottom left of Fig 3A. Since the eigenvectors of the effective connectivity matrix for homogeneous transfer functions are frequency independent, the mapping to the mode activity ${\tilde{R}}_{k}\left(\omega \right)$ is also constant across frequencies. The modes can be considered as decoupled circuits, whose activity is fed back to itself and can be treated in isolation. I.e. an adjustment of the connectivity of one mode does not influence the activity of another mode. The sum of activities in the circuit, however, is independent of the representation

$$R\left(\omega \right)=\sum_{i=1}^{N}R_{i}\left(\omega \right)e_{i}=\sum_{i=1}^{N}{\tilde{R}}_{i}\left(\omega \right)u_{i}^{A}.$$

The spectrum produced by the original circuit is given by the sum of the spectra generated by all possible mode pairs Eq (25)

$$C_{kk}\left(\omega \right)=\sum_{i,j=1}^{N}\beta_{ij}^{A}\left(\omega \right)T_{ij,k}^{A},\;k\in 1,..,N.$$ (29)

Here the spectrum visible in population *k* receives contributions from all mode pairs *i* and *j*. The prefactors *β*^*A*^_*ij*_(*ω*) are common to the spectrum of all populations and thus determine the global frequency dependence of the spectra. The visibility of the global characteristics of the spectrum in the spectra of the individual populations is determined by the frequency independent factor $T_{ij,k}^{A}$. The prefactor *β*^*A*^_*ij*_(*ω*) is large if one of the eigenvalues of the effective connectivity matrix comes close to one at a particular frequency *ω*_0_, resulting in a peak of the spectrum. Therefore, at peak frequency *ω*_0_ the contribution of the critical mode (*i* = *j* = *c*) constitutes the dominant part of the spectrum and we can approximate the spectrum of the circuit by the spectrum of the critical mode Eq (26)

$$C_{kk}\left(\omega_{0}\right)\approx \beta_{cc}^{A}\left(\omega_{0}\right)T_{cc,k}^{A}.$$ (30)

The anatomical sub-circuit responsible for the peak can now directly be deduced from the definition of $T_{cc,k}^{A}$ as the outer product of the eigenvectors of the critical mode. Removing the correlations induced by these connections (i.e. substituting the input provided by these connections with white noise) from the anatomical connectivity matrix $M^{A}\to M^{A}-\lambda_{c}^{A}u_{c}^{A}v_{c}^{A,T}$ removes the contributions of the critical mode (in particular the peak in the spectrum), but leaves contributions of the remaining modes to the spectrum unaltered.

The assumption of identical transfer functions of the populations entering the previous argument requires equal dynamic states of all populations. This in turn results in all populations displaying the same firing rates, which disagrees with experimental findings.

We therefore need to take population specific transfer functions into account, resulting in frequency dependent eigenvectors and eigenvalues and hence frequency dependent representations of the dynamical modes. The activity of one mode is now given by ${\tilde{R}}_{k}\left(\omega \right)=v_{k}^{T}\left(\omega \right)R\left(\omega \right)$

$$\begin{array}{ccc} {\tilde{R}}_{k}\left(\omega \right) & = & \lambda_{k}\left(\omega \right)\left({\tilde{R}}_{k}\left(\omega \right)+{\tilde{X}}_{k}\left(\omega \right)\right) \end{array}$$

as illustrated in the bottom right of Fig 3A.

In this case not only the prefactor but also the outer product of the eigenvectors is frequency dependent

$$C_{kk}\left(\omega_{0}\right)\approx \beta {}_{cc}\left(\omega_{0}\right)T_{cc,k}\left(\omega_{0}\right).$$ (31)

The peak in the spectrum and hence the dynamics of the critical eigenmode at *ω*_0_ could be removed by the adjustment $M\left(\omega \right)\to M\left(\omega \right)-\lambda_{c}\left(\omega \right)u_{c}\left(\omega \right)v_{c}^{T}\left(\omega \right)$. However, due to the frequency dependence of the mode representation, the same set of anatomical connections relevant for this mode at *ω*_0_ might also take part in the generation of the dynamics of another mode at a different frequency. Therefore removing the dynamical contribution of the anatomical connections contributing to one mode at one frequency will remove this particular oscillation, but may also impair other modes.

### Eigenvalue trajectories are transfer functions of the dynamical modes

The rates observed in population *k* are given by

$$R_{k}^{\text{pop}}\left(\omega \right)=e_{k}^{T}R\left(\omega \right)=\sum_{j=1}^{N}{\tilde{M}}_{d,kj}\left(\omega \right)\left(R_{j}^{\text{pop}}\left(\omega \right)+X_{j}^{\text{pop}}\left(\omega \right)\right)$$ (32)

with $X_{j}^{\text{pop}}\left(\omega \right)=e_{j}^{T}X\left(\omega \right)$ representing the noise projected into the direction of the *j*-th population. The rate of one dynamical mode is given by

$$R_{k}^{\text{mode}}\left(\omega \right)=v_{k}^{T}\left(\omega \right)R\left(\omega \right)=\lambda_{k}\left(\omega \right)\left(R_{k}^{\text{mode}}\left(\omega \right)+X_{k}^{\text{mode}}\left(\omega \right)\right)$$ (33)

with $X_{j}^{\text{mode}}\left(\omega \right)=v_{k}^{T}\left(\omega \right)X\left(\omega \right)$ representing the noise projected into the direction of the *k*-th mode. From the latter expression we conclude, that the eigenvalue trajectory acts as the transfer function of the dynamical modes. Considering Fig 4A and 4B we observe that the shape of the eigenvalue trajectory can roughly be approximated by a low pass filter with additional factors accounting for the mean delay and the delay distribution

$$\lambda_{k}\left(\omega \right)\approx \frac{\lambda_{k}\left(0\right)}{1+i\omega \tau_{k}^{\text{eff}}}e^{-i\omega d_{k}^{\text{eff}}}e^{-\frac{\sigma_{d_{k}^{\text{eff}}}^{2}\omega^{2}}{2}}.$$

The time constant $\tau_{k}^{\text{eff}}$, the delay $d_{k}^{\text{eff}}$, as well as the variance of the delays $\sigma_{d_{k}^{\text{eff}}}^{2}$ are effective parameters which, in multi-dimensional networks, are analytically intractable functions of the network parameters. Their definitions, however, suffice to gain an intuition for changes in synaptic parameters. The effective time constant determines the convergence speed of the trajectory towards zero. Hence larger effective time constants, potentially arising from large synaptic time constants, prevent the transmission of large frequencies. Larger effective delays accelerate the spinning of the trajectory, resulting in resonances at smaller frequencies, but also support rate instabilities in terms of Hopf bifurcations (see “*Stability of the dynamical modes*”) Larger variances of the effective delays compress the eigenvalue trajectory, resulting in smaller peaks in the spectrum. This effect of desynchronization of population dynamics by heterogeneity in the connection parameter has been demonstrated previously [87].

### Stability of the dynamical modes

To analyze the stability of the circuit, we consider the convolution equation, that describes the rates in a self-consistent manner, without noise

$$r_{i}\left(t\right)=\int_{-\infty }^{t}\sum_{j=1}^{N}M_{ij}^{A}H_{ij}\left(t-s\right)\;r_{j}\left(s-d_{ij}\right)\;ds=\sum_{j=1}^{N}M_{ij}^{A}H_{ij}*r_{j}\left(\circ -d_{ij}\right).$$ (34)

The variable describing the rate of each population can be replaced by its Laplace back-transformation

$$r_{i}\left(t\right)=\frac{1}{2\pi i}\int_{-i\infty }^{i\infty }e^{zt}R_{i}\left(-iz\right)\;dz$$ (35)

for complex *z*. Here *R*_*i*_(*ω*) is the Fourier transform of *r*_*i*_ evaluated at the complex Laplace frequency *z* = *iω*. Since convolutions simplify to multiplication in Laplace space we get

$$\begin{array}{c} \;\frac{1}{2\pi i}\int_{-i\infty }^{i\infty }dz\;\left(R_{i}\left(-iz\right)-\underset{}{\sum_{j}{\underset{︸}{H_{ij}\left(-iz\right)\;e^{-zd_{ij}}M_{ij}^{A}}}_{={\tilde{M}}_{ij}\left(-iz\right)}}R_{j}\left(-iz\right)\right)e^{zt}=0\\ \Rightarrow \left(1-\tilde{M}\left(-iz\right)\right)\;R\left(-iz\right)=0. \end{array}$$ (36)

This condition is fulfilled if either **R**(−*iz*) is an eigenvector $\hat{R}\left(-iz\right)$ of $\tilde{M}\left(-iz\right)$ with eigenvalue λ(− *iz*) = 1 or **R**(− *iz*) equals zero. The integration in Eq (35) can hence be rewritten as a sum over all solutions *z*′ ∈ *Z*′ for which $\tilde{M}\left(-iz^{\prime }\right)\hat{R}\left(-iz^{\prime }\right)=\hat{R}\left(-iz^{\prime }\right)$

$$\begin{array}{ccc} r_{i}\left(t\right) & = & \frac{1}{2\pi i}\int_{-i\infty }^{i\infty }e^{zt}R_{i}\left(-iz\right)\;dz=\frac{1}{2\pi i}\int_{-i\infty }^{i\infty }e^{zt}\sum_{z^{\prime }\in Z^{\prime }}\alpha_{z^{\prime }}{\hat{R}}_{i}\left(-iz^{\prime }\right)\delta \left(z-z^{\prime }\right)\;dz\\ & = & \frac{1}{2\pi i}\sum_{z^{\prime }\in Z^{\prime }}\alpha_{z^{\prime }}{\hat{R}}_{i}\left(-iz^{\prime }\right)e^{z^{\prime }t}, \end{array}$$

where *α*_*z*′_ are as yet undetermined constants that could be determined when tackling the inhomogenous problem. Expressed in Fourier domain we have *z* = *iω* so that *e*^*z*′*t*^ turns into *e*^*iω*′*t*^ and ${\hat{R}}_{i}\left(-iz\right)$ into ${\hat{R}}_{i}\left(\omega \right)$

$$r_{i}\left(t\right)=\frac{1}{2\pi i}\sum_{\omega^{\prime }\in \Omega^{\prime }}\alpha_{i\omega^{\prime }}{\hat{R}}_{i}\left(\omega^{\prime }\right)e^{i\omega^{\prime }t}=\frac{1}{2\pi i}\sum_{\omega^{\prime }\in \Omega^{\prime }}\alpha_{i\omega^{\prime }}{\hat{R}}_{i}\left(\omega^{\prime }\right)e^{(-ℑ\left(\omega^{\prime }\right)+iℜ\left(\omega^{\prime }\right))\;t}.$$ (37)

The transfer function *h*(*t*) and therefore the effective connectivity as well as the activity *r*_*i*_(*t*) are real valued functions in time domain. With the complex component in Fourier domain originating from the argument *ω*′, we conclude that $\tilde{M}\left(-\omega^{\prime }\right)={\tilde{M}}^{*}\left(\omega^{\prime }\right)$ has the eigenvector $\hat{R}\left(-\omega^{\prime }\right)={\hat{R}}^{*}\left(\omega^{\prime }\right)$ if $\omega^{\prime }\in R$. Thus *Ω*′ contains pairs of values $\omega_{+}^{\prime }=ℜ\left(\omega^{\prime }\right)+iℑ\left(\omega^{\prime }\right)$ and $\omega_{-}^{\prime }=-ℜ\left(\omega^{\prime }\right)+iℑ\left(\omega^{\prime }\right)$ for each *ω*′. Eq (37) can hence be written as:

$$r_{i}\left(t\right)=\frac{1}{\pi }\sum_{\omega^{\prime }\in \Omega_{+}^{\prime }}ℜ\left(\alpha_{i\omega^{\prime }}{\hat{R}}_{i}\left(\omega^{\prime }\right)e^{iℜ(\omega^{\prime })t}\right)\;e^{-ℑ(\omega^{\prime })t}.$$ (38)

with $\Omega_{+}^{\prime }$ containing all $\omega_{+}^{\prime }\in \Omega^{\prime }$. The equation above reveals that unstable modes exist if there is a solution with ℑ(*ω*′) < 0. In the context of the eigenvalue trajectories (Fig 4A) in the microcircuit one needs to investigate the solutions for the eigenvalues for complex frequencies *ω*. It turns out that all eigenvalue trajectories spiraling around the right side of the critical value one exhibit an unstable solution (λ(*ω*′) = 1 for ℑ(*ω*′) < 0) (see inset in Fig 4A) and vice versa. If the course of an eigenvalue trajectory is altered by changes in parameters (for example indegrees) such that it passes the value one for a real valued frequency (λ(*ω*′) = 1 with ℑ(*ω*′) = 0), the system would undergo a Hopf bifurcation. This can also be shown by mapping Eq (36) to the system discussed in [Eq. 2.8 in 88].
